# Tuning White-Light Emission of POSS-Based Fluorescent Hybrid Porous Polymers via Physical Blending for White LEDs

**DOI:** 10.3390/polym17182558

**Published:** 2025-09-22

**Authors:** Qiming Huo, Zhuo Lv, Shengyu Feng, Dengxu Wang, Hongzhi Liu

**Affiliations:** 1 National Engineering Research Center for Colloidal Materials & Key Laboratory of Special Functional Aggregated Materials, Ministry of Education, Shandong Key Laboratory of Advanced Organosilicon Materials and Technologies, School of Chemistry and Chemical Engineering, Shandong University, Jinan 250100, China; 202232256@mail.sdu.edu.cn (Q.H.); lvzhuowang@126.com (Z.L.); fsy@sdu.edu.cn (S.F.); liuhongzhi@sdu.edu.cn (H.L.); 2 Shandong WOSAI New Material Technology Co., Ltd., Weifang 262600, China

**Keywords:** light-emitting diodes, white light, polyhedral oligomeric silsesquioxane, fluorescent porous polymers

## Abstract

The development of a straightforward strategy for preparing organic fluorescent materials, fine-tuning white-light emission, and subsequently constructing white light-emitting diodes (LEDs) is of great significance. Herein, we report on the modulation of white-light emission and the fabrication of white LEDs using polyhedral oligomeric silsesquioxane (POSS)-based fluorescent hybrid porous polymers (HPPs) through simple physical blending. Two HPPs, namely HPP-1 and HPP-2, which emit blue and red light, respectively, were synthesized via the efficient Heck reactions of octavinylsilsesquioxane with 4,4′-dibromobiphenyl and 1,3,6,8-tetrabromopyrene. By physically doping of HPP-1 and HPP-2 in variable ratios in solvent suspensions, it was discovered that white-light emission is significantly influenced by the concentrations of the materials and the excitation wavelength. Similar findings were also observed in the solid-state physical doping. An ideal white light emission with a CIE coordinate of (0.33, 0.33) can be achieved when excited at 380 nm with a mass ratio of HPP-1 to HPP-2 of 1:2. Finally, the two HPPs were dispersed in polysiloxane matrices, and a white LED with a CIE coordinate of (0.42, 0.36) was obtained. The LED exhibited a color rendering index of up to 90 and a correlated color temperature of 2858 K, realizing warm white light emission. This simple and convenient white-light regulation strategy holds great promise for application in the development of novel white LEDs based on organic fluorescent porous materials.

## 1. Introduction

Semiconductor devices incorporating organic materials as the light-emitting layer constitute a crucial category of light-emitting diode (LED) devices [1,2,3]. Among their variants, white LEDs, which emit white light, play a pivotal role in both display and lighting technologies [4,5]. Compared with conventional LED lighting, white LEDs excited by UV LEDs have many advantages, including softer light emission, reduced glare, and a high color rendering index (CRI > 80), which closely approximates natural light and accurately reproduces the colors of objects [5]. These characteristics contribute to diminished visual fatigue, making white LEDs a healthier lighting option. A critical factor in the performance of white LEDs is the spectral regulation ability (e.g., adjustable color temperature) and stability of their light-emitting materials. These properties significantly influence the overall performance, cost-effectiveness, and application potential of the devices [2,6,7,8,9,10,11,12].

Currently, there are predominantly two material design approaches to achieve spectral tunability in white LEDs. The first approach involves the combination of red, green, and blue organic light-emitting materials in specific proportions [13]. By modulating the intensity ratio of each monochromatic light, continuous adjustment of the color temperature can be realized [14]. For example, a blue chip can be combined with organic yellow and orange fluorescent dyes, such as coumarin derivatives and rhodamine B [15,16,17]. In this configuration, the blue light excites the organic dyes to emit yellow light, which subsequently combines with blue light to produce white light. The second approach focuses on the development of single-system white light-emitting materials. By leveraging the intrinsic energy level structure of the materials or through doping modification, an adjustable white light spectrum can be directly output [18,19,20,21]. However, the former approach occasionally encounters the issue of poor lifetime matching among the three-color materials [22,23,24]. This disparity in lifetimes can lead to inconsistent performance over time. The latter is limited by the complexity of material molecular design. This complexity makes it difficult to simultaneously achieve wide spectral coverage and high luminous efficiency [25]. More importantly, the synthesis of most of the utilized organic materials in LEDs demands laborious and complex procedures. These convoluted processes result in high costs, which in turn impede the promotion and practical industrial applications of white LEDs. Therefore, there is a strong imperative to develop a straightforward strategy for the preparation of organic fluorescent materials, the modulation of white-light emission, and the subsequent construction of white LEDs. This would not only simplify the manufacturing process but also potentially reduce costs, facilitating the broader adoption of white LEDs in the industrial sector.

Recently, fluorescent porous polymers (FPPs), as an emerging class of fluorescent materials, have attracted specific attention because of the co-existence of porous and conjugated features within their structures. In these structures, the rigid porous networks can restrict the torsion of aromatic units, mitigate intermolecular π–π stacking, enhance exciton mobility, and consequently, boost the fluorescence activity [26,27]. Moreover, the conjugated structures and fluorescence in FPP materials can be readily adjusted, ranging from simple phenyl units to extended aromatic units, heterocyclic aromatic units, and large macrocycles. This tunability results in a full range of emission wavelengths. These features suggest that FPP materials hold great potential for fluorescent applications, such as sensing and photocatalysts [26,27,28]. However, the exploration of the application of these materials in white LEDs remains very limited [21,29].

**Scheme 1:**
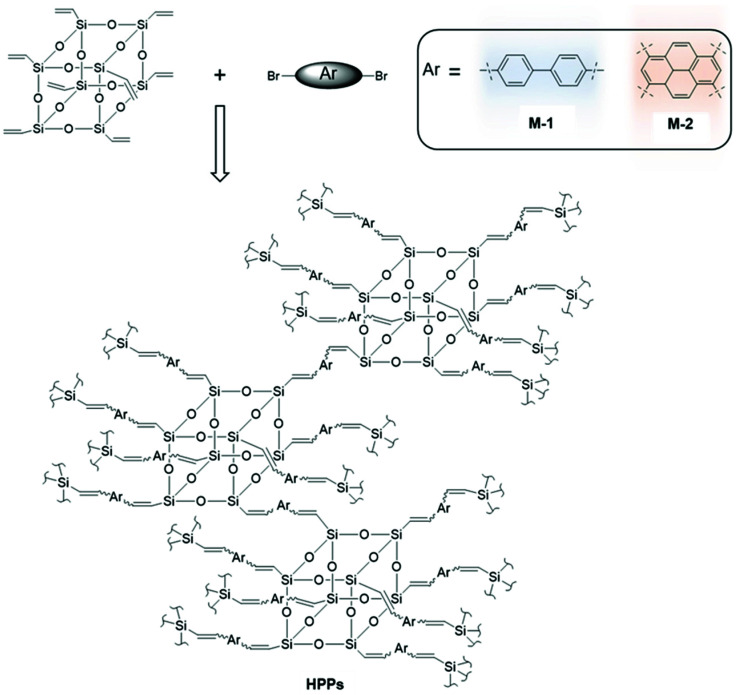
Synthetic routes of fluorescent hybrid porous polymers, HPP-1 and HPP-2. Reaction condition: Pd(PPh_3_)_4_, DMF/K_2_CO_3_, 120 °C, 72 h.

Herein, we report the utilization of polyhedral oligomeric silsesquioxane (POSS)-based fluorescent hybrid porous polymers (HPPs) in white LEDs. The blue light-emitting material HPP-1 and the red light-emitting material HPP-2 were synthesized via the Heck reactions of octavinylsilsesquioxane (OVS) with 4,4′-dibromobiphenyl and 1,3,6,8-tetrabromopyrene, respectively (Scheme 1). The choice of POSS-based HPP materials is motivated by their facile construction through the effective Heck reaction and the color-fluorescence tunability achieved by simply altering the monomer species, as demonstrated in our previous reports [30,31]. By means of simple physical doping of HPP-1 and HPP-2 in variable ratios through dispersion in solutions and solid-state powders, the fluorescence color of the mixed materials can be regulated, and white-light emission can be attained. Ultimately, the two HPPs were dispersed in polysiloxane matrices and coated on a UV LED substrate. Under the excitation of ultraviolet light, a warm white LED was obtained.

## 2. Materials and Methods

### 2.1. Materials

Unless otherwise noted, all reagents were purchased from commercial suppliers and used without further purification. N, N-Dimethylformamide (DMF), purchased from Shanghai Aladdin Biochemical Technology Co., Ltd. (Shanghai, China), was first dried over calcium hydride (CaH_2_) at 80 °C for 12 h, then distilled under reduced pressure and stored with 4 Å molecular sieves before use. Potassium carbonate, purchased from Shanghai Bide Pharmatech Ltd. (Shanghai, China), was dried in a vacuum drying oven at 100 °C for 12 h before use. Octavinylsilsesquioxane (OVS) was synthesized according to the method described in the previous report. The catalyst was purchased from Shanghai Bide Pharmatech Ltd. Ethanol, THF, methanol, and dichloromethane were purchased from Jinan Zheng Tao Instrument Co., Ltd. (Jinan, China). Fourier-transform infrared (FT-IR) spectra were acquired using a Bruker TENSOR-27 infrared spectrophotometer equipped with KBr particles, covering the range from 4000 cm^−1^ to 400 cm^−1^. Nitrogen adsorption isotherm measurements were carried out using a Micromeritics surface area and pore size analyzer. Before the measurement, the sample was degassed at 100 °C for at least 12 h. Nitrogen adsorption measurements at 77 K were performed using 100 mg of ultra-high-purity grade (99.999%) nitrogen gas from the Quadrasorb unit. Fluorescence spectra were determined using a Hitachi F-7100s fluorescence spectrophotometer with a monochromatic fluorescent lamp as the excitation source. The BET surface area was determined in the P/P_0_ range from 0.01 to 0.20, while the non-local density functional theory (NL-DFT) pore size distributions were analyzed using the carbon/slit-cylindrical pore mode of Quadrawin 2.0 software.

### 2.2. Synthesis of Fluorescent Hybrid Porous Polymers (HPPs)

The fluorescent hybrid porous polymers were synthesized using the procedures as described in our previous report [30].

Synthesis of HPP-1: Under an Ar atmosphere, octavinylsilsesquioxane (OVS, 632 mg, 1 mmol), Pd(PPh_3_)_4_ (63.2 mg, 0.054 mmol), potassium carbonate (2.20 g, 16 mmol), and N, N-dimethylformamide (DMF, 50 mL) were added to a 100 mL three-necked flask. After stirring at room temperature for 30 min, 4,4′-dibromobiphenyl (1248 mg, 4 mmol) was added. The resulting mixture was stirred at 120 °C for 48 h. After the reaction was completed, it was cooled to room temperature. The resulting mixture was filtered under suction, and the precipitation was washed with tetrahydrofuran, chloroform, water, methanol, and acetone to remove unconsumed monomers, inorganic salts, catalysts, and other residues. Using tetrahydrofuran and methanol as eluents, Soxhlet extraction was carried out for 24 h, respectively. Then, it was dried under vacuum at 70 °C for 48 h to obtain a solid powder of HPP-1 (1.023 g). Yield: 83%. Elemental analysis calcd (%) for HPP-1: C 62.3%, H 3.92%; found, C 60.04%, H 4.3%.

Synthesis of HPP-2: The synthesis steps of HPP-2 were same as those of HPP-1, except that 4,4′-dibromobiphenyl (1248 mg, 4 mmol) was replaced with 1,3,6,8-tetrabromopyrene (1036 mg, 2 mmol). Finally, a solid powder of HPP-2 (0.887 g) was obtained. Yield: 87%. Elemental analysis calcd (%) for HPP-2: C 56.44%, H 2.76%; found, C 53.45%, H 3.93%.

### 2.3. Light Modulation of HPP-1 and HPP-2 Dispersed in Solutions

First, 6 mg of dried HPP-2 was weighed into a round-bottom flask, followed by the addition of 20 mL of ethanol to prepare a suspension with a concentration of 0.3 mg/mL. Subsequently, 18 mg, 12 mg, 6 mg, 3 mg, and 2 mg of dried HPP-1 were added separately to prepare a series of mixed solutions with varying mass ratios, where the mass ratios of HPP-1 to HPP-2 were 3:1, 2:1, 1:1, 1:2, and 1:3, respectively. Ultrasonically disperse the mixtures for 20 min until a uniform suspension is formed. Fluorescence spectra were recorded under different excitation wavelengths (365 nm and 400 nm), and the corresponding color coordinates were calculated and plotted in the CIE chromaticity diagram. For HPP-2 suspensions with concentrations of 0.4 mg/mL and 0.5 mg/mL, the experimental procedures were identical to those described above, except for the adjusted masses of the fluorescent polymers.

When keeping the concentration of the HPP-2 suspension constant and also fixing the mass ratio of HPP-1 to HPP-2, different solvents are selected, such as polar solvents (methanol, ethanol, and tetrahydrofuran) and non-polar solvents (toluene, petroleum ether, and n-hexane). After ultrasonic dispersion to achieve a homogeneous state, the fluorescence spectra of the suspension are measured, and the CIE coordinates are calculated. Then, an analysis is carried out to determine whether there is a solvation effect in the light modulation in the solution.

### 2.4. Light Modulation of HPP-1 and HPP-2 in the Solid-State Powders

In total, 10 mg of dried HPP-1 powder was weighed and added to a flask containing 30 mL of ethanol. Subsequently, 5 mg, 10 mg, 20 mg, 30 mg, and 40 mg of dried HPP-2 powder were weighed separately and added to the flask to prepare suspensions with HPP-1 to HPP-2 mass ratios of 2:1, 1:1, 1:2, 1:3, and 1:4, respectively. The mixture was sonicated for 20 min to achieve uniform dispersion. Subsequently, the homogeneous suspension was subjected to rotary evaporation to yield a uniformly mixed solid powder of HPP-1 and HPP-2. Fluorescence spectra were tested under different mass ratios and excitation wavelengths, respectively, and the positions of the coordinate points in the CIE diagram were analyzed. Additionally, the light modulation behavior of the fluorescent hybrid porous materials in the solid powder state under different mass ratios was investigated.

### 2.5. Fabrication of LED Lamps Based on HPP-1 and HPP-2

Firstly, commercially available Dow Corning 184 silicone rubber was prepared as the encapsulating material. According to a mass ratio of 1:4, 1 g of Component A and 4 g of Component B were weighed and added to a round-bottom flask, followed by thorough mixing. Phosphor powder (50 mg), consisting of a pre-weighed mixture of HPP-1 and HPP-2, was then added to the mixed silicone rubber at a doping concentration of 1%. Based on the mass ratio of HPP-1 to HPP-2 (2:1), 33.3 mg of dried HPP-1 powder and 16.7 mg of dried HPP-2 powder were weighed separately and added to the flask (for a mass ratio of HPP-1 to HPP-2 of 1:4, 10 mg and 40 mg were weighed, respectively). The mixture was stirred uniformly with a glass rod, and a small amount of ethanol (approximately 10 mL) was added, followed by ultrasonic treatment to achieve uniform dispersion. The mixture was subjected to reduced-pressure rotary evaporation for 10 min to remove part of the solvent, and then transferred to a vacuum drying oven at 50 °C for 3 h to eliminate the residual solvent.

After the solvent was completely evaporated, the powder–glue mixture was dropped onto a 365 nm ultraviolet chip and cured in an oven at 100 °C for 1 h to complete the encapsulation. Subsequently, the photometric, colorimetric, and electrical properties (e.g., CIE coordinates and correlated color temperature) of the LED device were tested using a spectroradiometer equipped with an integrating sphere.

## 3. Results and Discussion

### 3.1. Synthesis, Porosity, and Fluorescent Properties of HPPs

**Figure 1:**
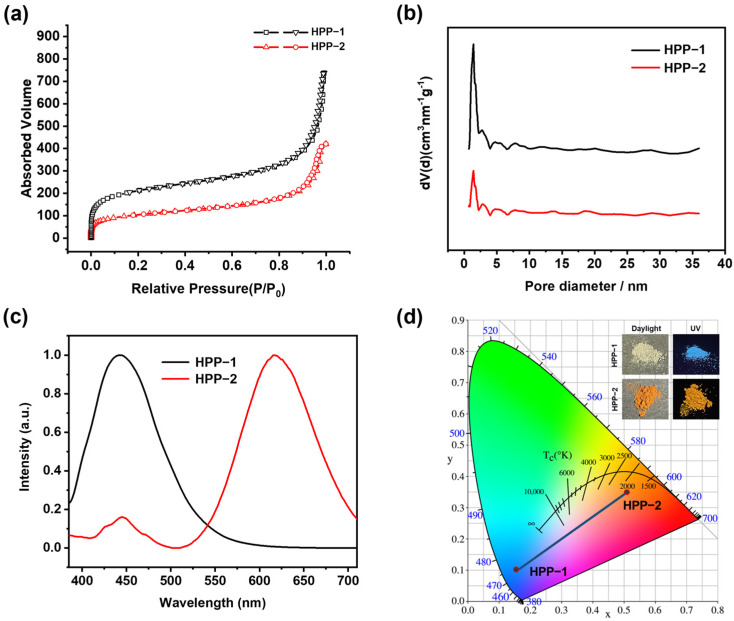
(**a**,**b**) Nitrogen adsorption and desorption isotherms (**a**) and pore size distribution curves (**b**) of HPP-1 and HPP-2; (**c**) Fluorescence emission spectra of HPP-1 and HPP-2 in ethanol solution (concentration: 0.1 mg/mL, excitation wavelength λ_ex_ = 365 nm); (**d**) CIE chromaticity diagram coordinate graph, and photos of HPPs under daylight and 365 nm ultraviolet light.

Two fluorescent hybrid porous polymers (HPPs), HPP-1 and HPP-2, were synthesized through the Heck reaction of octavinylsilsesquioxane (OVS) with 4,4′-dibromobiphenyl (M-1) and 1,3,6,8-tetrabromopyrene, respectively (Scheme 1), as previously reported in our work [30]. HPP-1 and HPP-2 were obtained as solids with light yellow and brick-red colors, respectively (inset in Figure 1d). The nitrogen adsorption and desorption isotherms measured at 77 K reveal that they exhibit similar sorption characteristics. There is a sharp increase in the uptake at low relative pressures, followed by a gradual increase in uptake at higher relative pressures with hysteresis. This behavior suggests the presence of both mesopores and micropores (Figure 1a). The Brunauer–Emmett–Teller surface areas (S_BET_) of HPP-1 and HPP-2 were determined to be 772 and 378 m^2^ g^−1^, respectively, and the total pore volumes (V_total_) were 0.80 and 0.65 cm^3^ g^−1^, respectively. Non-local density functional theory revealed that HPP-1 and HPP-2 possess a narrow distribution of micropores centered at ~1.4 and 1.7 nm, respectively, along with a broad distribution of mesopores centered at ~2.7 nm. This finding demonstrates the co-existence of micro- and mesopores (Figure 1b), which is consistent with the results from the nitrogen isotherms. The morphological images, observed by field emission scanning electron microscopy (FE-SEM), revealed that all polymers exhibit similar nanostructured pellets with a relatively uniform diameter of ca. 50 nm (Figure S1).

HPP-1 and HPP-2 are fluorescent and emit blue and red light, respectively, when exposed to UV light (inset in Figure 1d). The absolute fluorescence quantum yields of HPP-1 and HPP-2 in the solid state were 22.42% and 2.46%, estimated using the Wrighton−Ginley−Morse method. The fluorescence spectra of the suspensions of HPP-1 and HPP-2 in ethanol (0.1 mg/mL) are shown in Figure 1c. Upon excitation at 365 nm, HPP-1 exhibits a maximum fluorescence emission wavelength (λ_max_) at 443 nm. Its commission internationale de l’éclairage (CIE) coordinates are (0.15, 0.11) (Figure 1d), characteristic of a typical blue-light emission. In contrast, HPP-2 has a λ_max_ at 616 nm with CIE coordinates of (0.51, 0.36) (Figure 1d), which is indicative of a typical red-light emission. Notably, as shown in Figure 1d, the line connecting the chromaticity coordinates of HPP-1 and HPP-2 traverses the white light region. Therefore, theoretically, it is anticipated that white light emission can be achieved by compounding HPP-1 and HPP-2. Moreover, HPP-1 and HPP-2 exhibit relatively broad emission peaks in their fluorescence spectra. Consequently, LEDs prepared based on these two fluorescent porous polymers are likely to possess a high color rendering index, thus presenting warm white light emission.

As mentioned above in the introduction, the second approach for fabricating white LEDs involves the development of single-system white light-emitting materials. This is typically achieved by exploiting the intrinsic energy level structure of the materials or through doping modification. This approach is also referred to as the co-monomer doping strategy [29]. In our previous work [30], we attempted to apply this strategy to generate white light emission by simultaneously incorporating biphenyl and pyrene units into the porous networks. Unfortunately, this attempt was unsuccessful. However, we observed tunable fluorescence with a continuous color transition from blue to red when altering the molar ratio of biphenyl and pyrene units. Consequently, we explored the feasibility of the first strategy, which entails combining blue (HPP-1) and red (HPP-2) organic light-emitting materials in specific proportions through simple physical blending.

### 3.2. Tuning White-Light Emission by Physically Blending HPPs in Solvents

Firstly, HPP-1 and HPP-2 were physically blended at various ratios by dispersing them in solvents. Initially, the dispersion concentration of HPP-2 was fixed at 0.3 mg/mL. By adjusting the mass of HPP-1, the mass ratios of HPP-1 to HPP-2 were set to 3:1, 2:1, 1:1, 1:2, and 1:3, respectively. The influence of different mass ratios of the two materials on the fluorescence properties of the suspensions was investigated.

**Figure 2:**
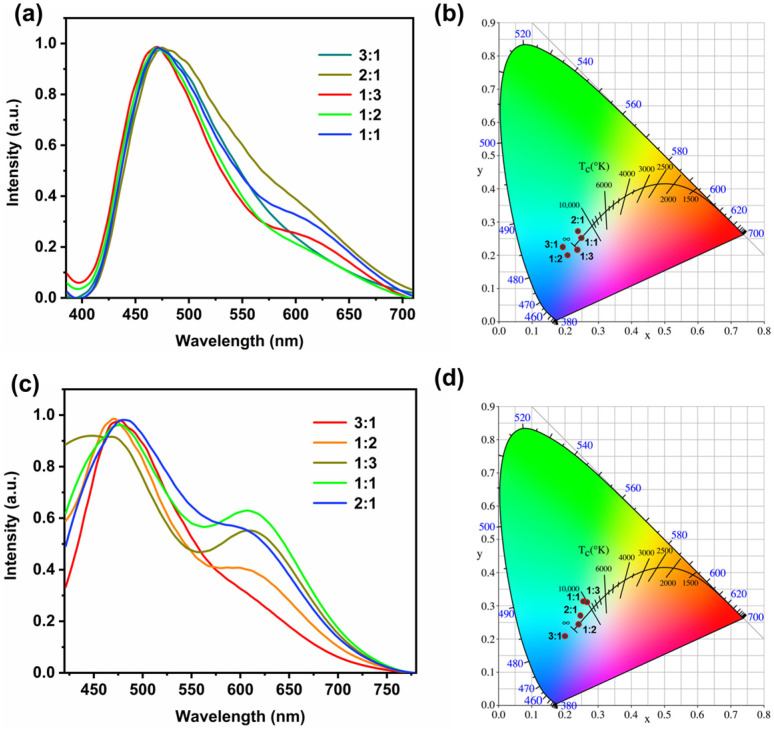
(**a**,**b**) Fluorescence emission spectra of HPP-1 and HPP-2 (**a**) and CIE chromaticity diagram coordinate graph (**b**) at different mass ratios (0.3 mg/mL in ethanol dispersion, λ_ex_ = 365 nm); (**c**,**d**) Fluorescence emission spectra of HPP-1 and HPP-2 (**c**) and CIE chromaticity diagram coordinate graph (**d**) at different mass ratios (0.3 mg/mL in ethanol dispersion, λ_ex_ = 400 nm).

As depicted in Figure 2a, under the excitation of 365 nm, as the proportion of HPP-2 gradually increased, the intensity of the red-light peak increased incrementally compared with the fluorescence intensity of the blue-emitting material HPP-1. When the mass ratios of HPP-1 to HPP-2 were 3:1, 2:1, 1:1, 1:2, and 1:3, respectively, the CIE coordinates were (0.20, 0.22), (0.24, 0.27), (0.24, 0.25), (0.21, 0.20), and (0.23, 0.22), respectively (Figure 2b and Table 1). This indicates that as the mass ratio of the red-emitting material HPP-2 increased, the CIE coordinates slightly shifted towards the red-light region.

According to previous reports [32,33,34], the fluorescence peak values change significantly under different excitation wavelengths. Therefore, we further examined the fluorescence spectra of different doping ratios under 400 nm excitation (Figure 2c). Compared with the fluorescence spectra under 365 nm excitation, the red-light peaks of HPP-2 in the fluorescence spectra corresponding to each ratio became more prominent. In the CIE diagram (Figure 2d), when the mass ratios of HPP-1 to HPP-2 were 3:1, 2:1, 1:1, 1:2, and 1:3, respectively, the CIE coordinates were (0.21, 0.21), (0.25, 0.28), (0.27, 0.30), (0.24, 0.25), and (0.27, 0.30), respectively (Table 1). The results demonstrate that as the excitation wavelength increases, the CIE coordinates after doping all shift to varying degrees towards the white-light region. When the mass ratio is 2:1, the CIE coordinates fall within the white light region, suggesting that white light emission can be achieved by adjusting the excitation wavelength.

**Figure 3:**
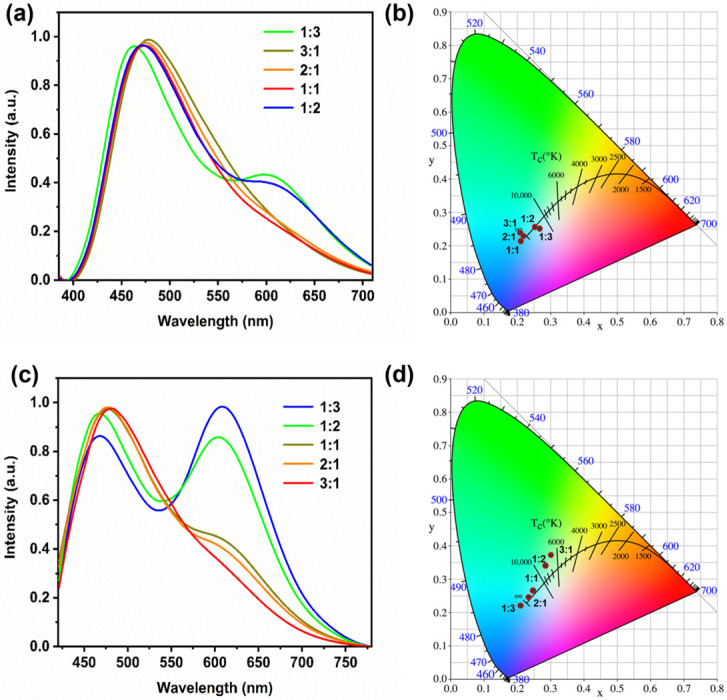
(**a**,**b**) Fluorescence emission spectra of HPP-1 and HPP-2 (**a**) and CIE chromaticity diagram coordinate graph (**b**) at different mass ratios (0.4 mg/mL in ethanol dispersion, λ_ex_ = 365 nm); (**c**,**d**) Fluorescence emission spectra of HPP-1 and HPP-2 (**c**) and CIE chromaticity diagram coordinate graph (**d**) at different mass ratios (0.4 mg/mL in ethanol dispersion, λ_ex_ = 400 nm).

Based on the aforementioned results, the concentration of HPP-2 was increased to 0.4 mg/mL and 0.5 mg/mL, respectively, to enhance the relative intensity of the red-light peak to the blue-light peak. When the concentration of HPP-2 was 0.4 mg/mL and the mass ratios of HPP-1 to HPP-2 were maintained at 3:1, 2:1, 1:1, 1:2, and 1:3, under the excitation of 365 nm, the fluorescence spectrum reveals that the fluorescence peak of HPP-2 was more distinct compared to that at a concentration of 0.3 mg/mL (Figure 3a).

The CIE coordinates were (0.21, 0.24), (0.22, 0.23), (0.21, 0.22), (0.25, 0.25), and (0.26, 0.25), respectively (Figure 3b and Table 1). These coordinates also shifted towards the white-light region, indicating that increasing the concentration of the red-emitting material can bring the emission closer to white light. Meanwhile, the fluorescence spectra and the CIE coordinates under 400 nm excitation were also measured when the concentration of HPP-2 was 0.4 mg/mL and the mass ratios of HPP-1 to HPP-2 were kept at 3:1, 2:1, 1:1, 1:2, and 1:3 (Figure 3c and Table 1). Analysis of the fluorescence spectra demonstrated that as the proportion of HPP-2 increased, the double peaks in the fluorescence spectrum became increasingly prominent. The CIE coordinates were (0.21, 0.22), (0.23, 0.24), (0.24, 0.25), (0.28, 0.34), and (0.30, 0.37), respectively (Figure 3d). These findings indicate that under these conditions, when the mass ratio of HPP-1 to HPP-2 was 1:2, the material exhibited superior white light emission.

**Figure 4:**
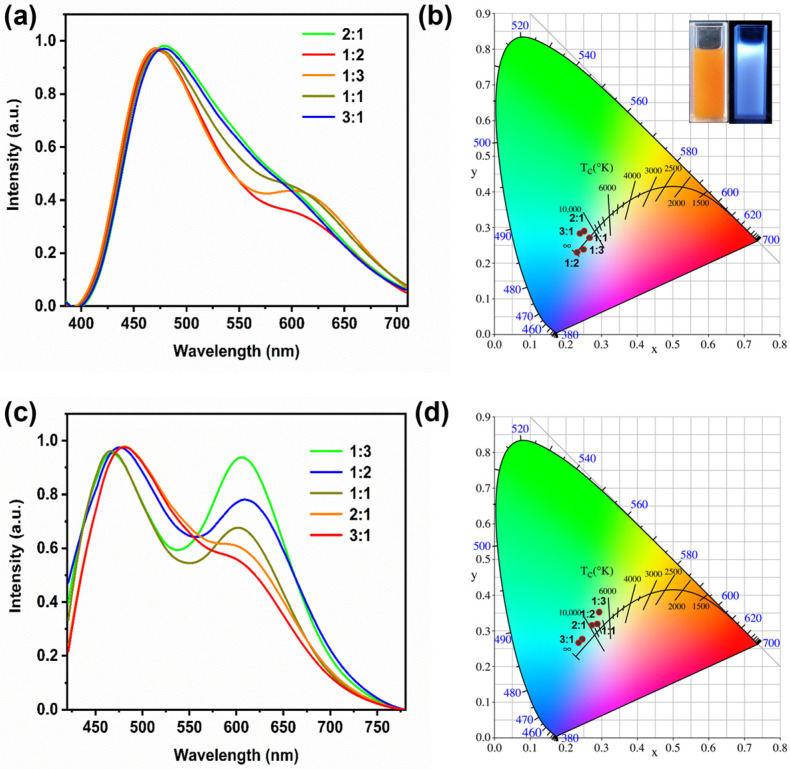
(**a**,**b**) Fluorescence emission spectra of HPP-1 and HPP-2 (**a**) and CIE chromaticity diagram coordinate graph (**b**) at different mass ratios (0.5 mg/mL in ethanol dispersion, λ_ex_ = 365 nm); The inset is the photograph of the mixture of HPP-1 and HPP-2 dispersed in ethanol at a mass ratio of 1:1. (**c**,**d**) Fluorescence emission spectra of HPP-1 and HPP-2 (**c**) and CIE chromaticity diagram coordinate graph (**d**) at different mass ratios (0.5 mg/mL in ethanol dispersion, λ_ex_ = 400 nm).

To further achieve white light emission under 365 nm excitation, we measured the fluorescence spectra with the concentration of HPP-2 set at 0.5 mg/mL while maintaining the mass ratios of HPP-1 to HPP-2 at 3:1, 2:1, 1:1, 1:2, and 1:3 (Figure 4a). The CIE coordinates were calculated to be (0.24, 0.27), (0.24, 0.28), (0.26, 0.27), (0.25, 0.25), and (0.26, 0.26), respectively (Figure 4b and Table 1). These findings indicate that under 365 nm excitation, the emission closest to the white light region was obtained at a mass ratio of 1:1. Meanwhile, the photograph taken under a 365 nm UV lamp also corroborated the obtained white light emission (inset in Figure 4b). White light emission was also realized at this concentration under 400 nm excitation. Based on the analysis of the fluorescence spectra (Figure 4c) and the CIE diagram (Figure 4d), for the fluorescence spectra corresponding to these ratios, consistent with the previously observed phenomena, the red-light peak became more pronounced. When the mass ratios of HPP-1 to HPP-2 were 3:1, 2:1, 1:1, 1:2, and 1:3, the CIE coordinates were (0.24, 0.27), (0.25, 0.28), (0.28, 0.32), (0.27, 0.32), and (0.29, 0.26), respectively (Table 1), and nearly all of them falls within the white light region.

**Figure 5:**
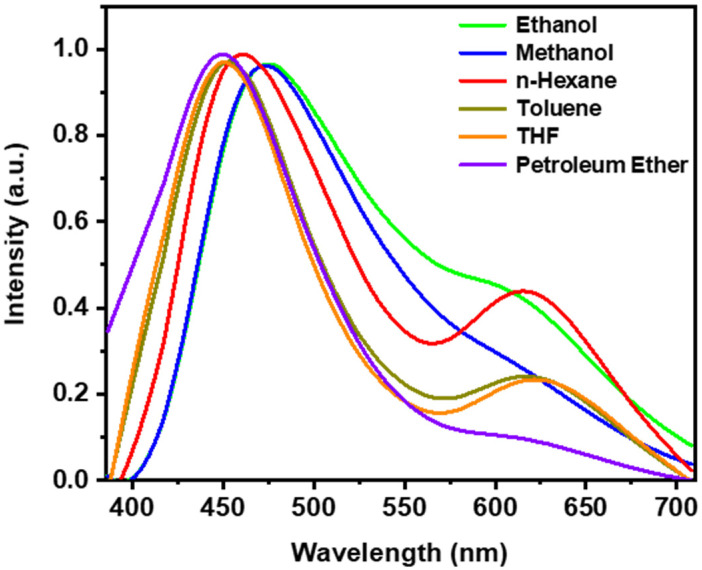
Fluorescence spectra of HPP-1 and HPP-2 in different solvents at a mass ratio of 1:1 (0.5 mg/mL).

Previous reports demonstrated that the polarity of organic solvents has often been found to influence the fluorescence emission wavelength of materials [35,36]. Consequently, we selected polar solvents (e.g., methanol and tetrahydrofuran) and non-polar solvents (e.g., toluene and petroleum ether). Under 365 nm excitation, with the concentration of HPP-2 at 0.5 mg/mL and the mass ratio of HPP-1 to HPP-2 maintained at 1:1, the fluorescence spectra of the materials in different solvents were measured (Figure 5). As shown in Table 2, the CIE coordinates in ethanol, methanol, tetrahydrofuran, toluene, petroleum ether, and n-hexane were (0.26, 0.27), (0.22, 0.24), (0.26, 0.21), (0.25, 0.21), (0.20, 0.15), and (0.28, 0.25), respectively. These results suggest that the fluorescence emission spectra of the materials do not exhibit a regular change with the alteration of solvent polarity, indicating the absence of an obvious solvation effect. The discrepancies in CIE coordinates may be due to the varying dispersibility of the materials in different solvents.

### 3.3. Tuning White-Light Emission by Physically Blending HPPs in the Solid Powder State

Based on the variations in the fluorescence spectra of the physical doping of HPP-1 and HPP-2 in solvents, white light emission can be obtained from the dilute solution dispersion of the materials. If such white light emission can be replicated in the solid state, especially in the form of a thin film, it is anticipated that these materials can be applied in lighting devices.

**Figure 6:**
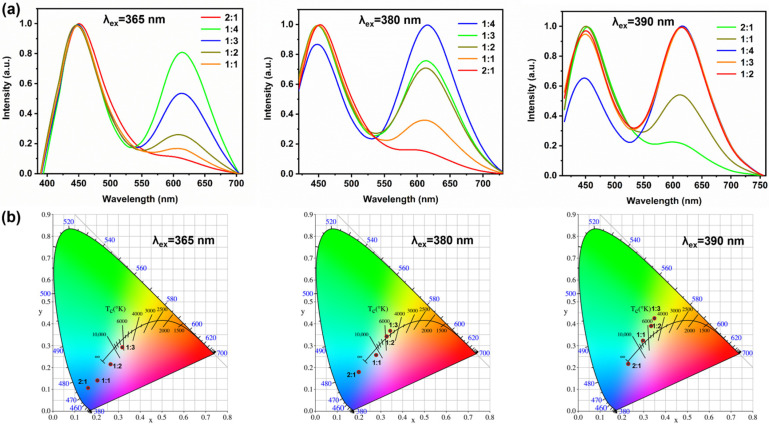
Fluorescence spectra (**a**) and CIE chromatograms (**b**) of HPP-1 and HPP-2 at different mass ratios in the solid state (λ_ex_ = 365 nm, λ_ex_ = 380 nm, λ_ex_ = 390 nm).

Consequently, we proceeded to explore the fluorescence emission of HPP-1 and HPP-2 in the solid powder state. When the mass ratios of HPP-1 to HPP-2 were 2:1, 1:1, 1:2, 1:3, and 1:4, respectively, we measured the fluorescence spectra under different excitation wavelengths of 365 nm, 380 nm, and 390 nm, along with the corresponding coordinates in the CIE diagram. As shown in Figure 6a, as the proportion of HPP-2 gradually increases, the red-light peak becomes more prominent compared to the blue-light peak. From the CIE diagram (Figure 6b), at a fixed excitation wavelength, the CIE coordinates of the solid powders doped with different mass ratios are distributed along a straight line.

According to the CIE coordinates summarized in Table 3, which correspond to different excitation wavelengths and mass ratios, emissions within the white light region were obtained. Under 365 nm excitation, when the mass ratio of HPP-1 to HPP-2 was 1:3, the CIE coordinate was (0.32, 0.29), which is very close to the ideal white light point (0.33, 0.33). When excited at 380 nm with a mass ratio of HPP-1 to HPP-2 of 1:2, the CIE coordinate was exactly (0.33, 0.33), representing an ideal white light emission. Similarly, when excited at 390 nm with a mass ratio of HPP-1 to HPP-2 of 1:1, the CIE coordinate was (0.30, 0.32). These results indicate that in the solid state, ideal white light emission can be readily obtained through physical mixing, thereby offering the potential for application in white LEDs.

**Figure 7:**
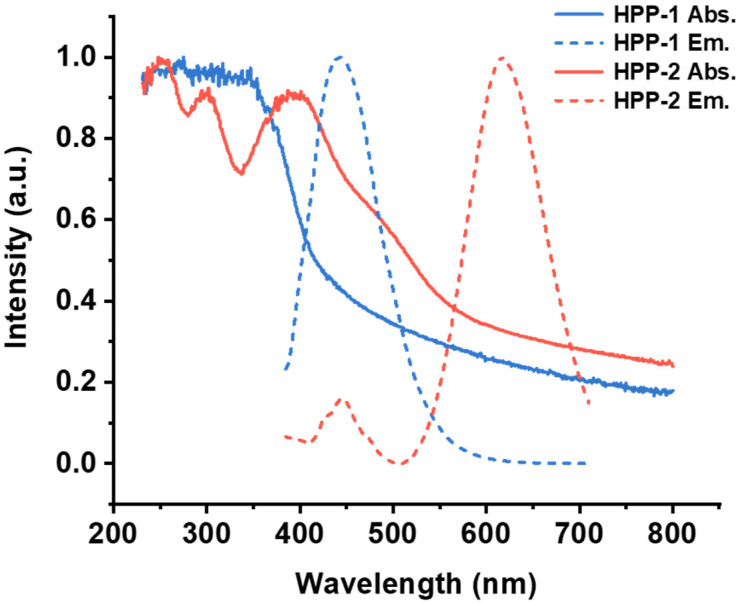
The normalized emission and absorption spectra of HPP-1 and HPP-2 in the solid state.

To investigate the mechanism of these color tunings and the generation of white light emission, the UV absorption spectra and emission spectra of HPP-1 and HPP-2 were measured. As illustrated in Figure 7, a significant spectral overlap (within the wavelength range of 421~484 nm) is observed between the emission spectrum of HPP-1 and the absorption spectrum of HPP-2. This overlap indicates that when HPP-1 emits fluorescence, HPP-2 can efficiently capture the fluorescent photons emitted by HPP-1 and subsequently emit fluorescence. Given the large distance and the absence of oriented dipole moments between HPP-1 and HPP-2, attributable to their porous structures, this mechanism is identified as reabsorption rather than Förster resonance energy transfer (FRET). Such a reabsorption effect provides the possibility for the generation of white light via the physical doping of HPP-1 and HPP-2.

This finding provides a plausible explanation for the significant shift in CIE coordinates observed with varying blend ratios and excitation wavelengths. As the blend ratio of HPP-2 increases, the number of HPP-2 molecules capable of absorbing the emission from HPP-1 increases proportionally. Consequently, the effective emission intensity of HPP-1 diminishes (due to reabsorption), while the emission of HPP-2 becomes relatively more dominant. Under different excitation wavelengths (e.g., 365 nm vs. 400 nm), the excitation efficiency of HPP-1 varies. A higher excitation efficiency of HPP-1 (e.g., at 400 nm) results in more emission that can be reabsorbed by HPP-2, thereby leading to a more significant shift in CIE coordinates (Figure 2b,d, Figure 3b,d and Figure 4b,d). In Figure 6b, as the excitation wavelength redshifts from 365 nm to 390 nm, the CIE coordinates shift toward the red-light region overall. These findings further confirm the effect of reabsorption.

### 3.4. Fabrication of White Light LED Lamps

**Figure 8:**
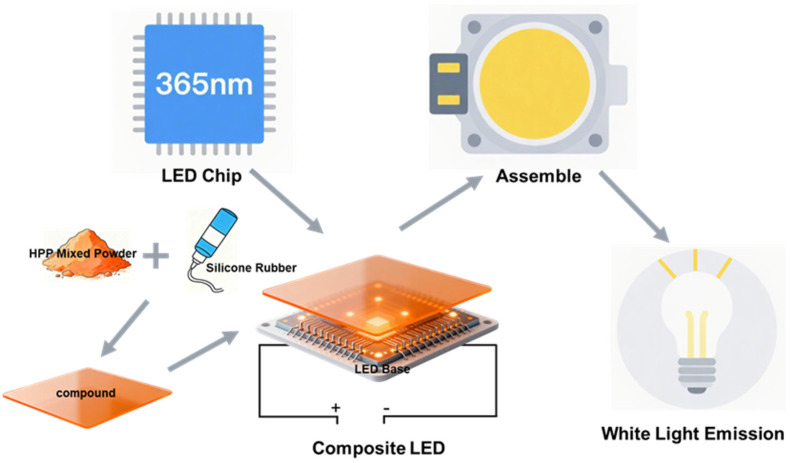
Schematic of the manufacturing process.

**Figure 9:**
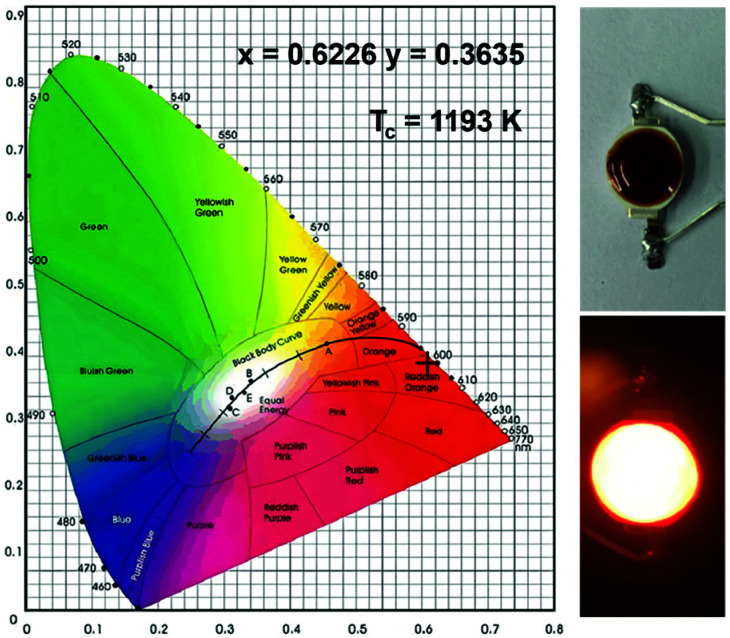
CIE chromaticity diagram of HPP-1 and HPP-2 when the mass ratio is 1:3, and the photo of the LED lamp.

Based on the excellent dimmability of HPP-1 and HPP-2 in the solid state, we employed these materials in the fabrication of white light LEDs by dispersing them in polysiloxane matrices. To ensure the universality of LEDs and achieve a high color rendering index, 365 nm ultraviolet UV LEDs were selected. In a typical procedure, the phosphor power, consisting of a pre-weighed mixture of HPP-1 and HPP-2, was incorporated into the Dow Corning 184 silicone rubber, which serves as the encapsulating material. Following uniform mixing of the powder and matrix, the mixture was dropped onto a 365 UV chip and cured, thereby fabricating LED devices (Figure 8). Additionally, given that the structure of the fabricated LEDs is very simple, the schematic of the composite LED in Figure 8 clearly depicts the cross-sectional composition. Specifically, the device consists of two layers: a UV LED chip and a compound layer composed of HPPs and silicon rubber. When the doping concentration was 1%, we utilized the blended powder with a mass ratio of HPP-1 to HPP-2 of 1:3, which was optimized through solid-state dimming, for the encapsulation and testing of the LED. As depicted in Figure 9, the CIE coordinate, measured by a spectrometer equipped with an integrating sphere, was (0.62, 0.36). This result indicates a substantial shift towards the red-light region following the doping and subsequent curing with silicone gels.

**Figure 10:**
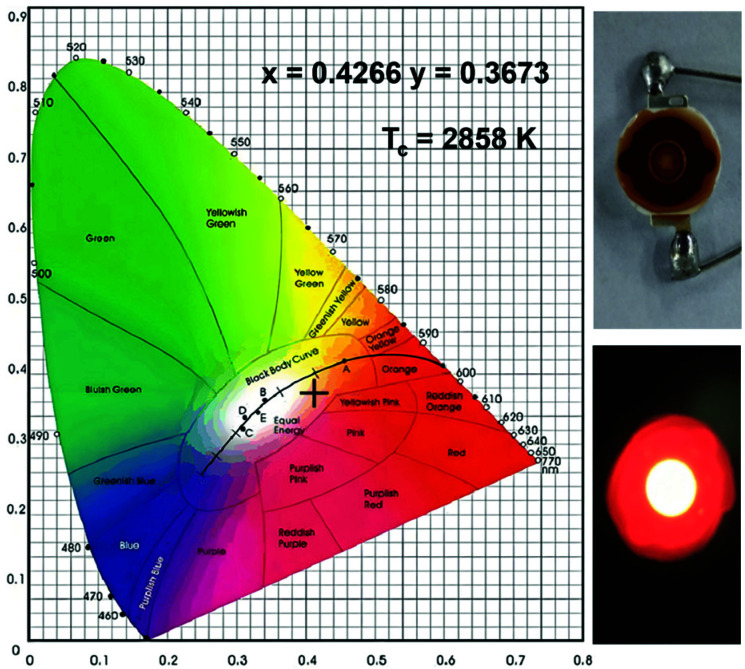
CIE chromaticity diagram of HPP-1 and HPP-2 when the mass ratio is 2:1, and the photo of the LED lamp.

By adjusting the mass ratios of HPP-1 and HPP-2, a white light LED was successfully fabricated when the mass ratio of HPP-1 to HPP-2 was 2:1. As illustrated in Figure 10, the CIE coordinate was (0.42, 0.36) and the correlated color temperature (CCT) was 2858 K, presenting a warm-white light emission, and the color rendering index (CRI) reached as high as 89.9. These results confirm the successful development of a white LED with a high CRI based on POSS-based fluorescent porous materials.

In comparison to the CIE coordinates of the ideal (0.33, 0.33) observed in powder blends (380 nm excitation, HPP-1:HPP-2 = 1:2), the CIE coordinates of the LED device exhibit a red-shift to (0.42, 0.36). This finding can be attributed to the UV absorption property of the Dow Corning 184 silicone rubber used as the encapsulation material. As depicted in Figure S2, this material exhibits characteristic UV absorption in the range of 200~380 nm, which encompasses the 365 nm excitation wavelength employed in the LED device. This absorption can reduce the effective UV energy reaching HPP-1, thereby diminishing its emission intensity. Consequently, the red emission from HPP-2, which was initially balanced by the emission of HPP-1, becomes relatively more dominant, leading to an overall shift of the CIE coordinates toward the red region.

In future work, we intend to design targeted experiments to mitigate the impact of Dow Corning 184 silicone. This will involve optimizing the silicone composition, adjusting the HPP-1:HPP-2 ratio to compensate for the absorption-induced emission loss, or modifying the device structure to improve UV energy utilization.

The performance of the current LED is comparable to that of previously reported white LEDs utilizing inorganic or organic fluorescent materials, such as SrAl_2_O_4_:Eu (L30) (CRI 88, CCT 2369 K) [37], Eu^2+^, Mn^2+^ doped (Ca, Sr)_9_Sc(PO_4_)_7_ (CSSPO) (CRI 88, CCT 3122 K) [38], and Cs_2_BaP_2_O_7_:0.01Eu^2+^ (CBPO:Eu) (CRI 73, CCT 4214 K) [39] (Table 4). Nevertheless, the present white LED falls short when compared to certain high-performance white LEDs. For example, white LEDs employing nitrides/nitrogen oxides (e.g., YAG, β-SiAlON, Cs_2_MP_2_O_7_) doped with rare earth ions (Ce^3+^, Eu^2+^, etc.) demonstrated outstanding performance, featuring a CRI of 92.6, a CCT of 4044 K, and more ideal CIE coordinates of (0.34, 0.32) [39]. White LEDs prepared by compounding multi-colored carbon dots have a CRI as high as 90 and a CCT of 3473 K [11]. This gap can be ascribed to the significantly higher quantum yields of the fluorescent materials in these high-performance LEDs, which are in the range of 60% to 96.5% [11,39,40,41,42], in contrast to the quantum yields of 22.42% for HPP-1 and 2.46% for HPP-2, respectively.

Although the current white LEDs trail behind many white LEDs, this strategy employed to construct white LEDs offers several advantages. Firstly, the preparation of HPPs is highly straightforward through the effective Heck reaction, and the starting materials are commercially available at a low cost. Secondly, the fluorescent properties (e.g., emission colors and quantum yields) of HPPs can be readily adjusted by varying the monomer species [30,31]. Thirdly, HPPs can be uniformly dispersed or mixed in solvents, powders, and polymer matrices, leading to good processing flexibility. In light of these characteristics, the device at present exhibits potential for deployment in scenarios with moderately demanding requirements for light quality and efficiency. Notably, it holds promise for utilization in low-cost indoor auxiliary lighting applications, such as cabinet lights and indicator lights. In the subsequent research, we will focus on expanding the application prospects of this HPPs-based luminescence system. Additionally, we will conduct further optimization of the HPPs doping ratio, adjust the device packaging structure, and explore compatibility with different UV LED excitation sources. These efforts aim to enhance the key performance parameters of white LEDs.

## 4. Conclusions

In summary, two fluorescent hybrid porous polymers, HPP-1 and HPP-2, emitting blue and red light, were synthesized from OVS and 4,4′-dibromobiphenyl or 1,3,6,8-tetrabromopyrene through the Heck reaction. HPP-1 and HPP-2 were employed to modulate the white-light emission via physically doping in both solvents and solid-state powders. The results demonstrated that white-light emission is markedly affected by the concentrations of the materials and the excitation wavelength. In ethanol suspensions, white-light emission can be achieved by adjusting the concentration ratio of HPP-1 and HPP-2 and the excitation wavelength. For example, when the ratio of HPP-1 to HPP-2 was 1:1 and the concentration of HPP-2 was 0.5 mg/mL, white-light emission with a CIE coordinate of (0.26, 0.27) was obtained under 365 nm excitation. In solid-state doping, ideal white-light emission with a CIE coordinate of (0.33, 0.33) was realized at an excitation wavelength of 380 nm with a mass ratio of HPP-1 to HPP-2 of 1:2. Subsequently, this strategy was utilized to fabricate white LEDs. The two materials with the optimized doping ratio were blended into polysiloxane matrices, resulting in white-light emission. The fabricated white LED device exhibited good performance, featuring a CIE coordinate of (0.42, 0.36), the CRI of 89.9, and the CCT of 2858 K, which are the characteristics of warm white-light emission. These findings highlight that POSS-based fluorescent HPPs possess important application value and broad development prospects in the field of white LEDs. Further research will focus on enhancing the fluorescent properties (e.g., quantum yields) of POSS-based HPPs and further improving the performance of white LEDs. Additionally, this work may open up new venues for the exploration of white LEDs through physical blending of fluorescent porous polymers.

## Figures and Tables

**Table 1:** CIE coordinates of HPP-1 and HPP-2 at different concentrations, with different ratios and different excitation wavelengths.

C_HPP-2_ (mg/mL)	λ_ex_ (nm)	3:1	2:1	1:1	1:2	1:3
0.3	365	(0.20, 0.22)	(0.24, 0.27)	(0.24, 0.25)	(0.21, 0.20)	(0.23, 0.22)
	400	(0.21, 0.21)	(0.25, 0.28)	(0.27, 0.30)	(0.24, 0.25)	(0.27, 0.30)
0.4	365	(0.21, 0.24)	(0.22, 0.23)	(0.21, 0.22)	(0.25, 0.25)	(0.26, 0.25)
	400	(0.21, 0.22)	(0.23, 0.24)	(0.24, 0.25)	(0.28, 0.34)	(0.30, 0.37)
0.5	365	(0.24, 0.27)	(0.24, 0.28)	(0.26, 0.27)	(0.25, 0.25)	(0.26, 0.26)
	400	(0.24, 0.27)	(0.25, 0.28)	(0.28, 0.32)	(0.27, 0.32)	(0.29, 0.36)

**Table 2:** CIE coordinates of HPP-1 and HPP-2 in different solvents at a mass ratio of 1:1 (0.5 mg/mL).

λ_ex_ (nm)	Ethanol	Methanol	THF	Toluene	Petroleum Ether	n-Hexane
365	(0.26, 0.27)	(0.22, 0.24)	(0.26, 0.21)	(0.25, 0.21)	(0.20, 0.15)	(0.28, 0.25)

**Table 3:** CIE coordinates of HPP-1 and HPP-2 at different excitation wavelengths with different mass ratios.

λ_ex_ (nm)	2:1	1:1	1:2	1:3	1:4
365	(0.19, 0.14)	(0.22, 0.17)	(0.26, 0.21)	(0.32, 0.29)	(0.35, 0.30)
380	(0.21, 0.17)	(0.28, 0.26)	(0.33, 0.33)	(0.35, 0.36)	(0.36, 0.37)
390	(0.23, 0.21)	(0.30, 0.32)	(0.34, 0.39)	(0.35, 0.42)	(0.36, 0.43)

**Table 4:** Performance parameters of some white light LED luminescent materials in the past five years.

Sample	CRI	CCT Values (K)	CIE	Material Type	Ref.
L30	88	2369	(0.35, 0.37)	Inorganic	[37]
CBPO: Eu	92.6	4044	(0.34, 0.32)	Inorganic	[39]
Ca_3_Gd(AlO)_3_(BO_3_)_4_:Tb/Eu	85.3	4329	(0.37, 0.38)	Inorganic	[40]
CD@SiO_2_	89.1	4850	(0.35, 0.37)	Inorganic QD	[41]
CSSPO	88	3122	(0.45, 0.44)	Inorganic	[38]
SrLa_2_Mg_2_W_2_O_12_:Mn	29.5	1159	(0.73, 0.267)	Inorganic	[43]
KSF/YAG	92.6	2716	(0.45, 0.40)	Inorganic	[44]
NaY_2_Ga_2_InGe_2_O_12_:Eu	93	4823	(0.35, 0.35)	Inorganic	[42]
FAPbBr:Nd	/	5263	(0.33, 0.36)	Organic–Inorganic	[45]
CTAB-PQDs	/	6000	(0.32, 0.33)	Inorganic	[12]
TADF/ExCi	95	/	(0.46, 0.43)	Organic–Inorganic	[46]
o/m/p-CDs	90	3473	(0.33, 0.36)	Organic	[11]
PS-GMA	95.2	5306	(0.35, 0.34)	Organic Polymer	[47]
POSS-based HPPs	89.9	2858	(0.42, 0.36)	Organic porous polymer	This work

## Data Availability

The data that support the findings of this study are available from the corresponding author, D. Wang, upon reasonable request.

## Supplementary Materials

The following supporting information can be downloaded at: https://www.mdpi.com/article/10.3390/polym17182558/s1, Figure S1: FE-SEM images of HPP-1 (a) and HPP-2 (b); Figure S2: UV absorption spectrum and physical image of Dow Corning 184 silicone elastomer sheet.
