# Supplementary material for: Tuning White-Light Emission of POSS-Based Fluorescent Hybrid Porous Polymers via Physical Blending for White LEDs
Source: Polymers (Basel). 2025 Sep 22;17(18):2558. doi: 10.3390/polym17182558 (PMC12473614; doi:10.3390/polym17182558)
Supplement: Supplementary file 1 [file polymers-17-02558-s001.zip › polymers-3868856-supplementary.pdf]

Supplementary Materials

Tuning White-Light Emission of POSS-Based Fluorescent Hybrid Porous Polymers via Physical Blending for White LEDs

Qiming Huo [1], Zhuo Lv [1], Shengyu Feng [1], Dengxu Wang [1,2,*] and Hongzhi Liu [1]

[1] National Engineering Research Center for Colloidal Materials & Key Laboratory of Special Functional Aggregated Materials, Ministry of Education, Shandong Key Laboratory of Advanced Organosilicon Materials and Technologies, School of Chemistry and Chemical Engineering, Shandong University, Jinan 250100, China
[2] Shandong WOSAI New Material Technology Co., Ltd., Weifang 262600, China
* Correspondence: dxwang@sdu.edu.cn

Table of Contents

Figure S1. FE-SEM images of HPP-1 (a) and HPP-2 (b)

Figure S2. UV absorption spectrum and physical image of Dow Corning 184 silicone elastomer sheet

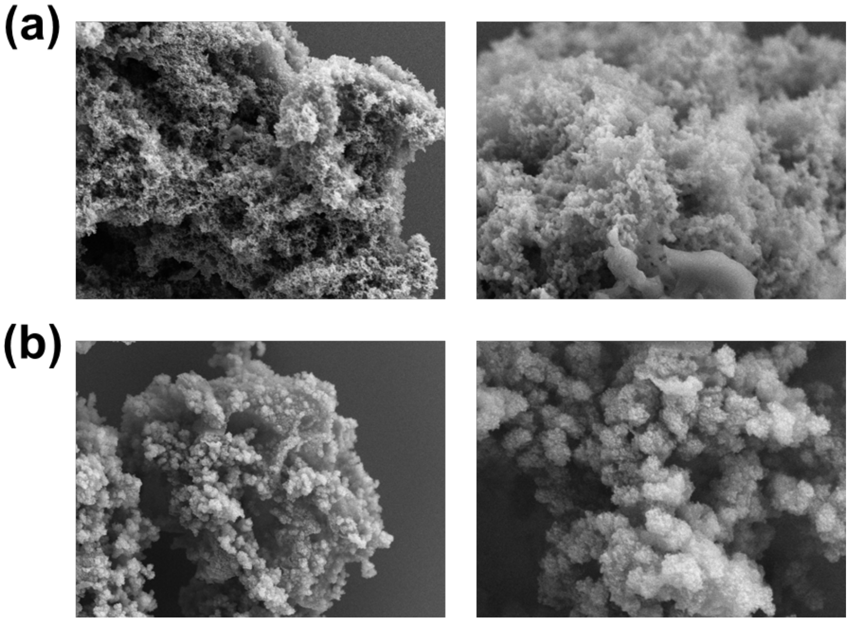

Figure S1. FE-SEM images of HPP-1 (a) and HPP-2 (b)

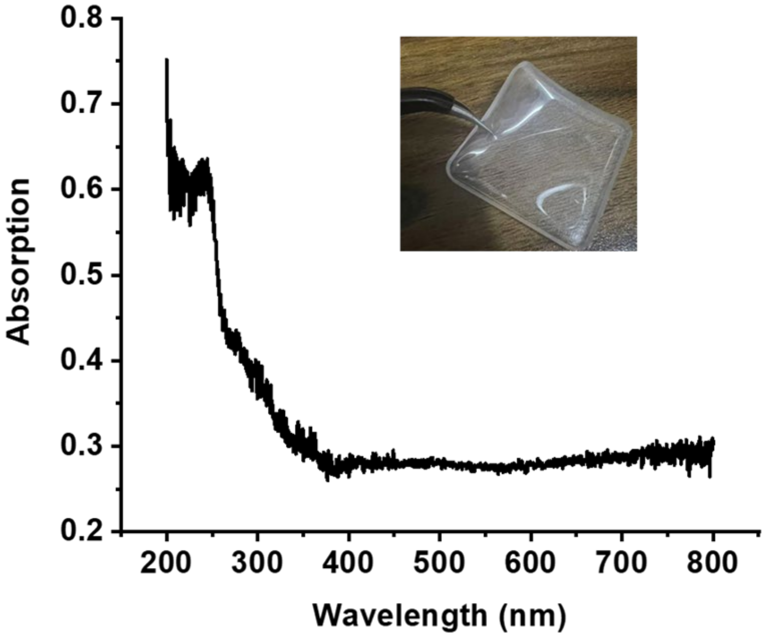

Figure S2. UV absorption spectrum and physical image of Dow Corning 184 silicone elastomer sheet
